# The psychosocial and mental health benefits of aquatic physical activity for individuals with disabilities: a systematic review from a sport psychology perspective

**DOI:** 10.3389/fpsyg.2026.1837063

**Published:** 2026-06-25

**Authors:** Ti Wu, Min-Hao Hung, Yen-Tzu Huang, Hsin-Huan Wang, Chun-Ju Liao, Po-Han Chang

**Affiliations:** 1 School of Physical Education, Sanming University, Sanming, China; 2 Office of Physical Education, National Chin-Yi University of Technology, Taichung, Taiwan; 3 Physical Education Office, National Cheng Kung University, Tainan, Taiwan; 4 Department of Tourism and Leisure Management, Yu Da University of Science and Technology, Zaoqiao, Taiwan; 5 Department of Athletic Sports, National Chung Cheng University, Chiayi, Taiwan; 6 Department of Athletics Sports, National Chung Cheng University, Chiayi, Taiwan

**Keywords:** adapted sports, aquatic physical activity, individuals with disabilities, mental health, sport psychology

## Abstract

**Purpose:**

Individuals with disabilities face a high risk of physical inactivity, leading to severe mental health challenges and social isolation. Aquatic physical activity, due to its unique physical properties, is considered an intervention with profound psychological potential. Guided by the International Classification of Functioning, Disability, and Health (ICF) framework and Self-Determination Theory (SDT), this study aims to systematically review the psychosocial, mental health, and functional benefits of aquatic physical activity for individuals with disabilities.

**Methods:**

This study strictly followed the PRISMA guidelines to search for high-quality literature published up to February 2026 in Web of Science, PubMed, Scopus, and SPORTDiscus. Two independent researchers conducted the screening based on strict PICOS criteria.

**Results:**

Following the search and screening, 21 core articles were included. The extracted data confirmed that aquatic interventions act as profound psychological facilitators. They significantly reduce depressive symptoms and anxiety (*Z* = −2.294) and enhance social skills and peer interaction (Cohen’s *d* = 0.76). Furthermore, physiological improvements (e.g., in gross motor function and balance) act as critical mediators that boost perceived competence and autonomy, effectively reducing kinesiophobia (fear of movement).

**Conclusion:**

Aquatic physical activity is a safe and highly effective adaptive health intervention. The aquatic medium serves as a “natural de-marginalisation space” that mitigates internal psychological barriers and societal ableism. These findings provide critical, evidence-based insights for sport psychology practitioners, coaches, and policymakers to design inclusive, confidence-building interventions.

## Introduction

Disability is a major global public health and human rights issue. According to the World Health Organization, approximately 15% of the global population lives with some form of disability (Alcaraz-Rodríguez et al., 2022). Due to physiological limitations, environmental barriers, and societal “ableism,” the physical activity (PA) participation rate of individuals with disabilities is significantly lower than that of the general population (Elipe-Lorenzo et al., 2025). This prolonged physical inactivity directly leads to severe secondary health conditions, most notably an extremely high prevalence rate of clinical depression, anxiety, and social exclusion (Jacob et al., 2023).

From a sport psychology perspective, the barriers to physical activity participation for this population are not merely physical. Research indicates that internal psychological barriers—such as fear of injury, lack of perceived competence, and the internalization of societal stigma—often outweigh external physical constraints (Ascondo et al., 2023; Olasagasti-Ibargoien et al., 2023). According to Self-Determination Theory (SDT), fulfilling the basic psychological needs of autonomy, competence, and relatedness is crucial for initiating and sustaining sport participation (Liu et al., 2025).

Land-based sports environments often fail to provide these psychological safe spaces due to high joint impact and the fear of falling. Therefore, “Aquatic Physical Activity” has garnered high attention as an alternative intervention. The aquatic environment possesses unique physical properties: buoyancy significantly offsets gravity, while hydrostatic pressure provides a natural, safe training environment (Mohamed et al., 2025). A recent scoping review by Ogonowska-Slodownik et al. (2024) corroborated that aquatic therapy has widespread positive impacts on rehabilitation and psychological wellbeing.

However, a critical gap remains in the sport psychology literature. Previous reviews focusing on aquatic interventions have primarily investigated isolated physiological outcomes, neglecting the profound psychological mechanisms at play. There is a distinct lack of comprehensive reviews that synthesize how these aquatic environments specifically foster mental health, psychological resilience, and social integration. Furthermore, existing studies lack a cohesive theoretical foundation to explain how physical improvements in the water translate to enhanced psychological wellbeing.

To address these gaps, this systematic review is firmly guided by the ICF framework and Self-Determination Theory (SDT). The purpose of this study is to systematically evaluate the psychosocial and mental health benefits of aquatic physical activity for individuals with disabilities across all life stages, providing impactful evidence for sport psychology practitioners and policymakers.

## Methodology

### Search strategy

This systematic review strictly adhered to the PRISMA 2020 guidelines (Page et al., 2021). The literature search was conducted across four major international databases: Web of Science, PubMed/MEDLINE, Scopus, and SPORTDiscus. To ensure the inclusion of the most contemporary evidence, the search timeframe covered January 2014 through February 2026.

### Eligibility criteria and PICOS framework

The inclusion criteria were defined using the PICOS framework. The population (P) comprised individuals with diagnosed disabilities across the lifespan. The intervention (I) included aquatic-based physical activities, such as adaptive swimming, aquatic board sports, and other adapted aquatic exercise programs. Comparison conditions (C) involved standard care, land-based exercise interventions, or pre–post baseline assessments. The primary outcomes (O) focused on psychosocial and mental health indicators, including depression, anxiety, social interaction, psychological resilience, motivation, and related psychological constructs. Secondary outcomes included physiological and functional measures, such as balance and motor function, which may contribute to psychological wellbeing through improvements in perceived competence and autonomy. The study design (S) included quantitative experimental studies, quasi-experimental studies, and high-quality qualitative syntheses that explored the psychological and functional effects of aquatic physical activity in individuals with disabilities.

### Study selection and quality assessment

Literature screening was independently conducted by two researchers, adhering to a person-first philosophy (using “participants” rather than “subjects”). Three distinct appraisal tools were utilized: the PEDro Scale for RCTs, the Downs and Black Scale for non-randomized interventions, and the CASP Checklist for qualitative studies.

## Results

### PRISMA flow diagram summary

**Figure 1:**
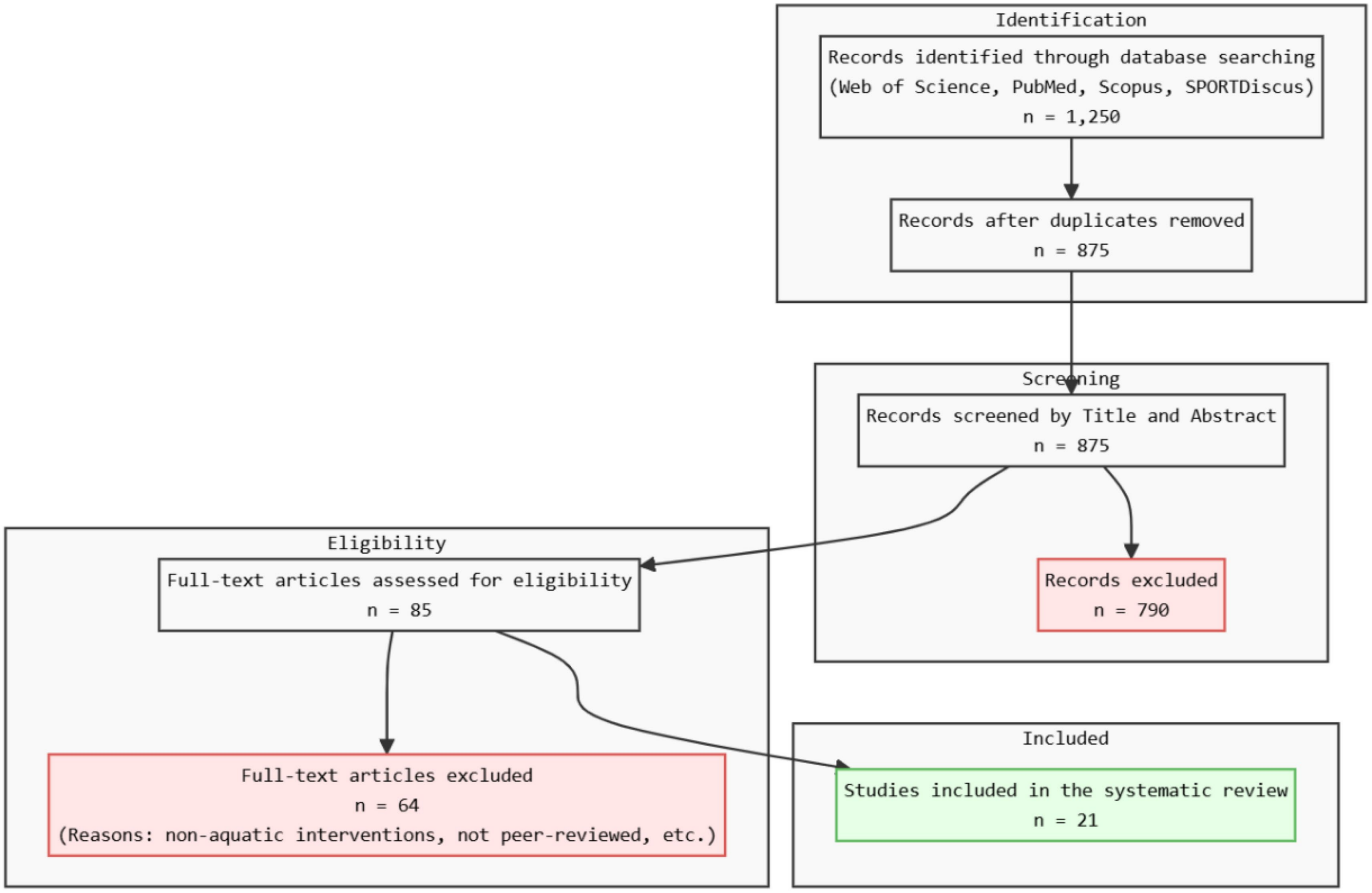
PRISMA flow diagram.

Through the updated search, 1,250 articles were initially retrieved. After screening and applying strict PICOS criteria, a total of 21 core SCI/SSCI articles were finally included. The overall risk of bias was low to moderate (see Figure 1, PRISMA Flow Diagram).

### Psychological and social benefits (primary outcomes)

In the realm of mental health and sport psychology, aquatic and physical activity interventions demonstrate profound efficacy. A comprehensive meta-analysis by Jacinto et al. (2023) confirmed that physical activity has a statistically significant and large effect on reducing depressive symptoms among individuals with disabilities (Effect size *Z* = −2.294, *p* = 0.022). This is further corroborated by Zhang et al. (2026), whose recent meta-analysis demonstrated that exercise significantly alleviates anxiety and psychiatric comorbidities in pediatric populations.

Regarding social skill development, Jeong and Kim (2025) revealed that PA significantly enhances social skills (overall Cohen’s *d* = 0.76), with peer-interactive interventions yielding the best results (*ES* = 1.15). Mu et al. (2025) highlighted that sports participation elevates self-esteem and mitigates social stigma, while Al Harthy et al. (2025) established a strong positive correlation between active sports club participation, social inclusion, and improved psychological wellbeing. Additionally, recent 2026 qualitative assessments by Mueller et al. (2026) demonstrate that participation in adaptive sports builds psychological resilience and fosters a “recovery mindset.”

### Physiological improvements as mediators of psychological competence

Physical improvements in the aquatic environment serve as the foundational mediators for building psychological confidence. Regarding pediatric populations, Mohamed et al. (2025) conducted a high-quality RCT implementing aquatic core stability training for children with cerebral palsy. The experimental group’s Gross Motor Function Measure (GMFM) and Pediatric Balance Scale (PBS) scores improved significantly (*p* < 0.001). From a sport psychology perspective, these physiological gains directly reduce the fear of falling and rapidly increase self-efficacy and perceived competence.

## Discussion

This systematic review, grounded in recent high-quality evidence through 2026, precisely evaluated the holistic benefits of aquatic physical activity. By synthesizing findings through the lens of Sport Psychology, SDT, and the ICF framework, the evidence cohesively demonstrates that aquatic interventions act as a powerful psychological catalyst.

### Aquatic environments as a catalyst for psychological empowerment (SDT perspective)

Applying Self-Determination Theory (SDT), the aquatic environment uniquely satisfies the basic psychological needs for competence, autonomy, and relatedness (Liu et al., 2025). The hydrostatic pressure and buoyancy provide a safe, anti-gravity environment that eliminates the fear of falling—a major psychological barrier and source of anxiety noted by Olasagasti-Ibargoien et al. (2023). Because participants can execute movements in the water that they cannot perform on land, they rapidly build perceived *competence* and *autonomy*. This psychological empowerment directly fuels intrinsic motivation to sustain physical activity (Table 1).

**Table 1:** Comprehensive summary of the 21 included studies.

No	First author (Year)	Study design	Participants	Intervention/Focus	Key findings
1	Al Harthy et al. (2025)	Cross-sectional Survey *(Downs & Black: Good)*	People with disabilities in sports clubs.	Association between social inclusion and mental health.	Established a strong positive correlation between active sports participation and psychological wellbeing.
2	Alcaraz-Rodríguez et al. (2022)	Systematic Review *(CASP: High)*	Individuals with visual impairment.	Influence of PA on social inclusion.	Physical activity serves as a primary vehicle for social normalization and peer-support.
3	Ascondo et al. (2023)	Cross-sectional Survey *(Downs & Black: Fair)*	Adults with various disabilities.	Barriers and motives for practicing PA.	Internal psychological barriers (fear, anxiety) outweigh external constraints.
4	Carretti et al. (2023)	Narrative Review *(CASP: High)*	Visually impaired individuals.	PA to improve balance control.	Demonstrated that PA provides proprioceptive stimulation to compensate for visual deficits.
5	Elipe-Lorenzo et al. (2025)	Systematic Review *(CASP: High)*	Individuals with disabilities in mainstream sports.	Physical and social barriers.	Identified “ableism” and lack of adaptive psychological support as main barriers to inclusion.
6	Jacinto et al. (2023)	Meta-Analysis *(Downs & Black: Good)*	Individuals with depressive symptoms.	Impact of PA on depression.	Exercise significantly reduced depressive symptoms (*Z* = −2.294). Confirmed PA as a primary psychological treatment.
7	Jacob et al. (2023)	Systematic Review *(CASP: High)*	Adults with intellectual disabilities.	PA benefits for Quality of Life.	Regular PA significantly promotes adaptive behaviors and enhances long-term quality of life.
8	Jariono et al. (2025)	Literature Review *(PRISMA: High)*	Children with special needs.	Impact of Adaptive PE on cognitive development.	Adapted sports significantly enhance executive functions and brain neuroplasticity.
9	Jeong and Kim (2025)	Meta-Analysis *(RoBANS: High)*	Individuals with disabilities across all ages.	Effects of PA on social skills.	Social skills significantly improved (*d* = 0.76); peer interaction showed the highest effect (*ES* = 1.15).
10	Judge et al. (2025)	Scoping Review *(CASP: High)*	Athletes with physical disabilities.	Adaptive synergy and enhancing inclusivity.	Highlighting how “Adaptive Synergy” fosters inclusivity and psychological motivation for all populations.
11	Li et al. (2025)	Mini-Review *(CASP: High)*	Individuals needing rehabilitation.	Combat sports in VR for disability adaptation.	Utilizing Virtual Reality to decrease initial kinesiophobia and improve rehabilitation adherence.
12	Liu et al. (2025)	Qualitative Meta-synthesis *(CASP: High)*	Students with disabilities in integrated schools.	Barriers and facilitators to PA.	Based on SDT, the lack of psychological competence is a core barrier requiring environmental adaptations.
13	Luarte-Rocha et al. (2025)	Systematic Review *(CASP: High)*	People with visual impairments.	Effects of team-sport practice.	Team-sport practice significantly improves self-esteem, wellbeing, and overall quality of life.
14	Mohamed et al. (2025)	RCT *(PEDro: High)*	Children with mild cerebral palsy.	Aquatic core stability and balance training.	GMFM improved to 94.82; PBS increased to 52.3 (*p* < 0.001). Physical gains mediate psychological autonomy.
15	Mu et al. (2025)	Systematic Review *(PRISMA: High)*	Disabled athletes.	Impact of sports on mental health.	Sports participation significantly elevates self-esteem, mitigates anxiety, and combats social stigma.
16	Mueller et al. (2026)	Qualitative *(CASP: High)*	Participants in adaptive sports programs.	Adaptive sport as holistic health intervention.	Adaptive sports build psychological resilience, self-efficacy, and a sustainable “recovery mindset.”
17	Ogonowska-Slodownik et al. (2024)	Scoping Review *(PRISMA: High)*	Children and adolescents with disabilities.	Aquatic therapy interventions.	Corroborated that aquatic therapy has widespread positive impacts on pediatric psychological and physical rehabilitation.
18	Olasagasti-Ibargoien et al. (2023)	Systematic Review *(CASP: High)*	Women with physical disabilities.	Barriers to physical activity.	Identified double vulnerability (gender and disability) and fear of injury as primary psychological deterrents.
19	Shen et al. (2024)	RCT *(PEDro: High)*	Adolescents with intellectual disabilities.	Adapted PA program based on the ICF framework.	Experimental group showed significant improvements in fundamental motor skills and psychosocial Quality of Life.
20	Tsai et al. (2020)	Observational *(PEDro: Moderate)*	Adult participants.	EMG comparison of aquatic paddling postures.	Kneeling and standing both highly activated core muscles, proving high postural adaptability.
21	Zhang et al. (2026)	Meta-Analysis *(PRISMA: High)*	Children with autism spectrum disorder.	Effects of exercise on anxiety.	Exercise significantly reduced anxiety and psychiatric comorbidities, showcasing robust mental health benefits.

### The “natural de-marginalisation space” and social integration

A central psychological finding synthesized from recent literature is the conceptualization of the aquatic environment as a “natural de-marginalisation space.” On land, mobility aids (wheelchairs, crutches) often act as visual markers that trigger societal “ableism” and internal stigmatization (Elipe-Lorenzo et al., 2025). Once an individual enters the water, these terrestrial rules disappear. Wheelchairs are left at the poolside, stripping away the visible symbols of disability. This environmental equalization neutralizes physical differences, dramatically reducing the perception of social marginalization. It fosters genuine peer interaction and *relatedness*, explaining the massive effect size (*ES* = 1.15) in social skill development observed during aquatic group activities (Jeong and Kim, 2025).

### Bridging the body–mind connection: operationalising the ICF framework

To fully understand the psychological and functional benefits of aquatic physical activity, the findings can be interpreted through the domains of the ICF framework. At the level of Body Functions and Structures, the unique physical properties of water facilitate improvements in core muscle activation, dynamic balance, and motor function, providing a foundation for enhanced physical competence (Mohamed et al., 2025). Within Activities and Participation, aquatic environments enable individuals with disabilities to perform movements that may not be feasible on land, thereby increasing engagement in meaningful activities and social participation. These experiences are associated with improvements in social skills, peer interaction, and community inclusion (Luarte-Rocha et al., 2025). Regarding Environmental Factors, aquatic settings function as supportive facilitators that reduce physical and psychological barriers to participation. Nevertheless, limited access to trained adaptive coaches and inclusive aquatic programs remains an important environmental constraint (Elipe-Lorenzo et al., 2025). Finally, Personal Factors appear to play a critical role in psychological adaptation. Through successful participation and skill mastery, individuals often develop greater self-efficacy, perceived competence, and autonomy, fostering a shift from a passive care-oriented identity toward a more empowered athletic self-concept. Such psychological gains may contribute to reduced depressive symptoms (*Z* = −2.294) and the development of a resilient recovery-oriented mindset (Mueller et al., 2026).

### Implications for sport psychology practitioners, coaches, and policymakers

From a sport psychology perspective, these findings provide several actionable implications for practice and policy. Aquatic physical activity should be recognized as an effective psychological health intervention that can complement traditional rehabilitation and mental health services for individuals with disabilities. The unique properties of water create opportunities for successful movement experiences, which may reduce anxiety, depression, and fear of movement while enhancing psychological wellbeing. Furthermore, coaches and educators should move beyond a purely performance-oriented approach and adopt strategies that promote autonomy, competence, and relatedness. By creating supportive aquatic learning environments and providing achievable challenges, practitioners can strengthen intrinsic motivation and long-term participation. At the policy level, investment in inclusive aquatic infrastructure, adaptive equipment, and professional training programs is essential to ensure equitable access to aquatic sport and physical activity. Such initiatives can facilitate social inclusion and help establish sustainable pathways for lifelong participation in sport among individuals with disabilities.

### Limitations and future directions

While the synthesized evidence is strong, this review acknowledges methodological limitations, such as the inability to blind participants in aquatic RCTs. Furthermore, as digital health technologies advance, future sport psychology interventions should explore the integration of Virtual Reality (VR) (Li et al., 2025). Utilizing VR as a preliminary mental exposure tool could effectively diminish hydrophobia and anxiety in first-time aquatic participants, opening a novel interdisciplinary frontier.

## Conclusion

This systematic review confirms that aquatic physical activity offers profound psychosocial and mental health benefits for individuals with disabilities. Viewed through the lens of Sport Psychology, SDT, and the operationalised ICF framework, the aquatic medium functions as a “natural de-marginalisation space”—removing terrestrial barriers, satisfying the basic psychological needs for autonomy and competence, and neutralizing the stigma of disability. Aquatic exercise stands as a validated, powerful prescription to combat depression, alleviate anxiety, and rebuild psychological resilience. Sport psychologists, coaches, and policymakers must collaborate to prioritize universal design in aquatic facilities, placing mental health and psychological empowerment at the forefront of adaptive sports.
